# A CASE STUDY: THE INVISIBLE LABOR OF CULTURALLY SENSITIVE CARE

**DOI:** 10.1093/geroni/igz038.2574

**Published:** 2019-11-08

**Authors:** Mai See Thao, Odichinma C Akosionu, Heather Davila, Tetyana P Shippee

**Affiliations:** 1 Medical College of Wisconsin, Milwaukee, United States; 2 University of Minnesota, Minneapolis, Minnesota, United States; 3 VA Boston Healthcare System, Boston, Massachusetts, United States

## Abstract

Nursing homes are increasingly becoming more racially/ethnically diverse yet racial disparities in resident’s quality of life and quality of care continue to persist. One reason for these disparities is lack of culturally-sensitive care and racial/ethnic similarity between residents and staff. This study examines a case of a high proportion minority nursing home with racially/ethnically diverse staff to understand how shared culture among direct care staff and residents may influence care delivery. We used three months of participant observation, supplemented by in-depth qualitative interviews with 8 Hmong residents and 5 Hmong staff to explore the labor of culturally sensitive care in a large, urban NH. We discovered four themes: 1) Culturally sensitive care was often equated to fulfilling language needs for residents who didn’t speak English. 2) Hmong staff members had to take the initiative to inform non-Hmong staff members how to care for Hmong residents. 3) Hmong staff members also had to communicate the culture of NH care and its limitations to Hmong residents and their families. 4) Hmong staff members have to advocate for the culturally relevant needs of Hmong residents. The findings of this case study illuminate that having staff members from diverse cultural backgrounds and meeting language needs of residents does not reflect the everyday practices of culturally sensitive care. This type of emotional labor can also result in higher levels of burn-out for staff of color. Additional research into what constitutes culturally sensitive care to NH residents and staff is needed.

